# Improvement of Dietary Diversity and Attitude toward Recommended Feeding through Novel Community Based Nutritional Education Program in Coastal Kenya—An Intervention Study

**DOI:** 10.3390/ijerph17197269

**Published:** 2020-10-05

**Authors:** Mami Hitachi, Violet Wanjihia, Lilian Nyandieka, Chepkirui Francesca, Norah Wekesa, Juma Changoma, Erastus Muniu, Phillip Ndemwa, Sumihisa Honda, Kenji Hirayama, Mohammed Karama, Satoshi Kaneko

**Affiliations:** 1 Program for Nurturing Global Leaders in Tropical and Emerging Communicable Diseases, Graduate School of Biomedical Sciences, Nagasaki University, Nagasaki 852-8523, Japan; hitachimami@gmail.com; 2 Centre for Public Health Research, Kenya Medical Research Institute (KEMRI), Nairobi 20752-00202, Kenya; vwanjihia@gmail.com (V.W.); nyandieka.n@gmail.com (L.N.); muniue@yahoo.com (E.M.); pndemwa@hotmail.com (P.N.); 3 Department of Community Health and Epidemiology, School of Public Health, Kenyatta University, Nairobi 43844-00100, Kenya; chepfran@gmail.com; 4 Kenya Medical Research Institute, KEMRI graduate school, Nairobi 54840-00200, Kenya; namemekesa@gmail.com (N.W.); mkarama@umma.ac.ke (M.K.); 5 NUITM-KEMRI Project, Institute of Tropical Medicine (NUITM), Nagasaki University, Nairobi 19993-00202, Kenya; jtasa06@gmail.com; 6 Department of Nursing, Graduate School of Biomedical Sciences, Nagasaki University, Nagasaki 852-8523, Japan; honda@nagasaki-u.ac.jp; 7 Department of Immunogenetics, Institute of Tropical Medicine, Nagasaki University, Nagasaki 852-8523, Japan; hiraken@nagasaki-u.ac.jp; 8 Department of Eco-epidemiology, Institute of Tropical Medicine, Nagasaki University, Nagasaki 852-8523, Japan

**Keywords:** education, intervention, child nutrition, dietary diversity, attitude, community health workers, small neighborhood units, Nyumba Kumi

## Abstract

Community-based nutritional intervention to improve the practice of dietary diversity and child nutrition by community health workers (CHWs) involving Nyumba Kumi as small neighborhood units (SNUs) in communities has not yet been explored. This study was conducted in two villages in rural Kenya between 2018 and 2019. In total, 662 participants (control vs. intervention: *n* = 339 vs. *n* = 323) were recruited. The intervention group received education on maternal and child nutrition and follow-up consultations. The custom-tailored educational guidelines were made based on Infant and Young Child Feeding and the mother and child health booklet. The educational effects on household caregivers’ feeding practice attitude and child nutritional status were analyzed using multiple linear regression. After the intervention, a total of 368 household caregivers (187 vs. 181) and 180 children (113 vs. 67) were analyzed separately. Between the groups, no significant difference was found in their background characteristics. This study successfully improved the dietary diversity score (β = 0.54; *p* < 0.01) and attitude score (β = 0.29; *p* < 0.01). The results revealed that the interventions using CHWs and SNUs were useful to improve dietary diversity and caregivers’ attitudes toward recommended feeding. This research has the potential to be successfully applied in other regions where child undernutrition remains.

## 1. Introduction

Undernutrition associates with nearly half of the deaths among children under the age of five, which means approximately three million young lives are unnecessarily lost in the world [1]. Furthermore, it increases the morbidity risks from several diseases among young children in the short term; in the long-term, it causes stunting and impaired cognitive development, which results in poor school performance and social development [2]. Although several efforts and measures have been taken to improve the nutritional status among children worldwide, it is still estimated that twenty-one percent of children still have a stunting condition or chronic undernutrition, especially in South Asia and sub-Saharan Africa [2,3,4]. Rich dietary diversity is an essential component to reduce undernutrition among children; however, less than 30% of children in sub-Saharan Africa are fed a “minimally acceptable” diet based on the global guidelines [5,6,7,8].

Kenya is one of 34 countries experiencing the highest burden of child undernutrition [9]. Approximately one in four children (26.2%) is estimated to be stunted in Kenya [10]. Tanaka et al. reported that the traditional diet pattern had low dietary diversity in their research in Kenya, and children fed by the traditional diets had a higher risk of stunting compared with those who were fed by non-traditional diet patterns [11]. Since dietary diversity for children depends on caregivers’ knowledge, attitude, and practice [12], it is essential to provide caregivers enough information about the appropriate feeding of children, including dietary diversities by health staff members during antenatal and postnatal care. However, those opportunities to receive such information are limited due to inadequate access to health facilities [13]. 

Under such limited situations to access health facilities, it is proven that community-based educational programs using community health workers (CHWs) improve the nutritional status of children in low-income and middle-income countries [9]. However, the effect on dietary diversity by a community-based approach with CHWs has not been well investigated. Although intervention with CHWs can have the potentiality to improve diet diversity, it would be challenging because of the CHWs’ low motivation, community supports for the CHWs, and cooperation with existing community organizations [14].

Additionally, there is another community-based structure, “Nyumba Kumi,” small neighborhood units (SNUs), in Kenya, which means ten households in the Swahili language. It is a mechanism introduced to achieve political stability and shared values in 2013 in Kenya by the presidential order. It is the smallest social unit located at the lowest governmental community policing structure [15,16]. The combined activities of CHWs and Nyumba Kumi might have synergistic effects regarding health education in the community; however, there has been no study on this matter to date.

This study aimed to prove the hypothesis that community-based nutritional educational programs cooperating with CHWs and Nyumba Kumi might effectively change the attitudes of caregivers toward feeding practices and dietary diversity for their children.

## 2. Materials and Methods

### 2.1. Pre-Intervention

**Figure 1:**
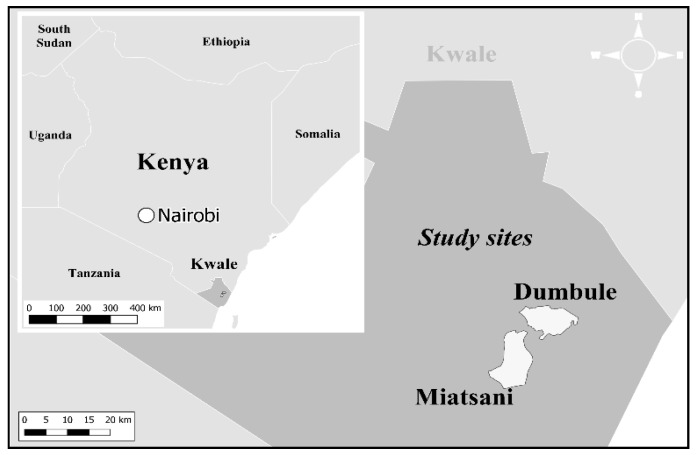
Map of the study site.

**Figure 2:**
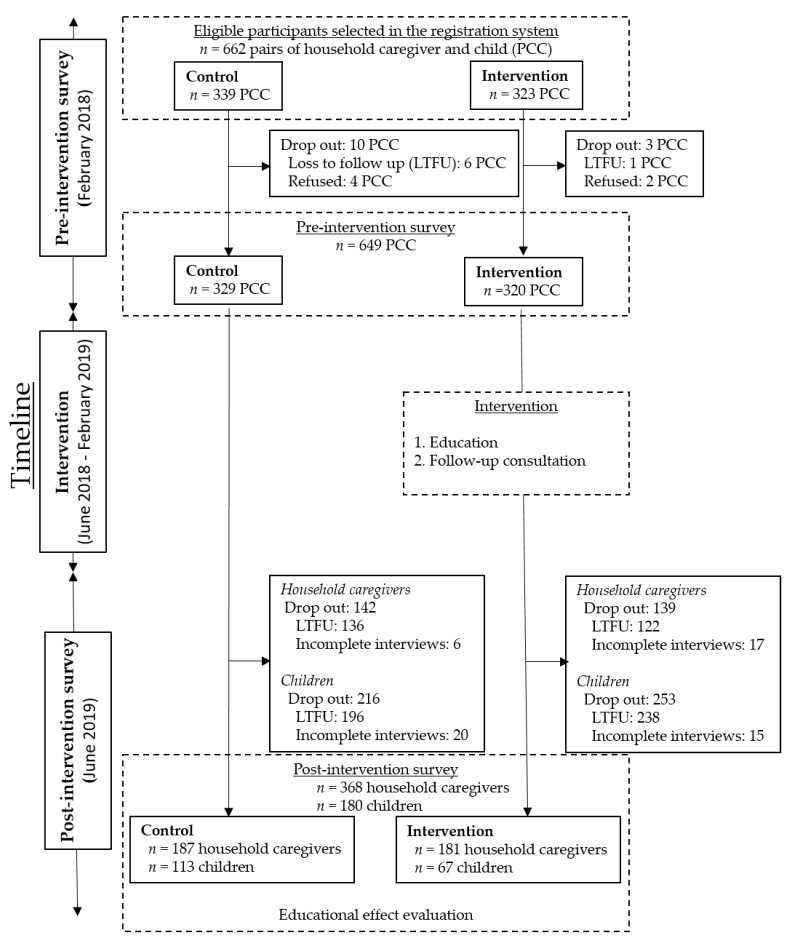
Flow-diagram of enrolled participants and survey procedure. PCC: pairs of caregivers and children; LTFU: lost to follow-up meaning participants were absent at the time of the survey; Incomplete interviews: data contained missing or contradictive values.

This study was conducted in the Kwale district of Kenya, where a Health and Demographic Surveillance System (HDSS) is being supervised under Nagasaki University and Kenya Medical Research Institute (Figure 1). The HDSS monitors the population dynamics every three months, and the system covers nearly 43,000 individuals in an area of 390 square km that includes ten villages [17]. Of the ten villages, two villages, Dumbule and Miatsani, were selected and assigned to a control and an intervention group. These two villages were chosen because of the similar backgrounds in terms of (i) climate settings, (ⅱ) ethnicity (Durumas) and culture, and (ⅲ) healthcare services (health facility and staff). Furthermore, we considered the geographical distance (30 km) to avoid intervention information “cross-over” from the intervention group to the control group. For the participant selection, 662 potential participants (control: *n* = 339; and intervention: *n* = 323) were recruited from the HDSS records. The study period was between February 2018 and June 2019 (Figure 2).

### 2.2. Intervention

Based on the pre-intervention survey, we found 649 pairs (control: *n* = 329; and intervention: *n* = 320) of caregivers and children who were potentially eligible for the educational intervention trial. The subjects were children aged 6–59 months and their caregivers (Figure 2). The trial had two groups: (i) control: caregivers did not receive any education on maternal and child nutrition, and (ii) intervention: caregivers received the education between June and August 2018, and a follow-up consultation was done between September 2018 and February 2019. The educators were locally recruited qualified nutritionists, and they were trained to follow the custom-tailored educational guidelines based on the WHO recommended indicators of Infant and Young Child Feeding and instructions written in the mother and child health booklet [18]. The custom-tailored guidelines contained general information on maternal and child nutrition and health, considering local situations and traditional practices: (i) maternal nutrition during pregnancy and lactation, (ii) early initiation of breastfeeding, (iii) exclusive breastfeeding and duration of breastfeeding and expressing breast milk, (iv) age-appropriate complementary feeding, (v) diverse diet and food groups, (vi) proper hygiene and sanitation practices, (vii) nutrient components of foods and supplements under the maternal and child health program, and (viii) malaria and soil-transmitted helminth and family planning. The education intervention was given in the local language using teaching aids like charts, pamphlets, and models to promote participants’ uptake of the materials. During the follow-up consultation, either a CHW or a member of the SNU visited the households in the intervention group, and they observed the households according to the list to confirm the practice they learned during the educational session. If their practices were improper, suggestions were provided as part of the educational program to follow the educational guidelines by discussing challenges and problems.

### 2.3. Data Collection

Our data collectors, a CHW, or a member of the SNU who was given training in advance, administrated a structured questionnaire survey at each caregiver’s household once in the pre- and post-intervention in February 2018 and June 2019, respectively. The training sessions were given and qualified by the trained nutritionists to keep the quality of the surveys. The questionnaire was designed to investigate household status (socioeconomic and demographic variables), household caregiver situation (attitudes toward recommended feeding and diet practice), and child status (age, sex, weight, and height). For the socioeconomic status (SES), the possession or use of the following contents were asked as a binomial variable (0/1): bedrooms (<2 or 2≤); cooking fuel (firewood or other); home electrical appliances (either phone, refrigerator, or T.V. set); mobile phone; bicycle; cart; car; and house (owner-occupied or not). The SES was divided into lower and higher statuses using a median threshold of SES. Besides, the household population, questions about religion (Islam or others), and delivery place (home or facility) were added to the demographic variables. The household population was categorized into two groups (<5 or 5≤) based on the average household population in Kwale county [19]. As asked in the household caregiver situation section in the questionnaire, caregiver attitude was assessed based on nine items corresponding to the tailored educational guidelines, and each item was scored one if the caregiver agreed (Table S1, Supplementary Materials). This attitude score ranged from zero to nine. The diet quality and food consumption in the previous 24 h were also asked of each caregiver. After the interview, food items were classified into 15 categories based on the FAO guidelines [20]: starchy staples (grains, white tubers, and roots); vitamin A-rich vegetables (vitamin A-rich vegetables and tubers); dark green leafy vegetables; other vegetables; vitamin A-rich fruits; other fruits; legumes and nuts; dairy products (milk, yogurt, and cheese); eggs; fresh meats; organ meats; fish and seafood; oil; sweets; and spices. Based on the classification, each category was binomially classified (0/1) and the sum was calculated (a.k.a. dietary diversity score (DDS)). The DDS ranged between zero and 15, with the higher score indicating higher diversity. Moreover, the body weights of children were measured using a digital scale (Seca Gmbh & Co.Kg, Hamburg, Germany), and heights were scaled using a UNICEF length measure (available online: https://www.unicef.org/supply/documents/height-length-measuring-boards). Both anthropometric scales were taken twice, and averages were recorded to minimize measurement errors in the field. Z-scores for height-for-age (HAZ), weight-for-age (WAZ), and weight-for-height (WHZ) were calculated based on the mean according to the Child Growth Standards published by the WHO in 2006 to evaluate child nutritional status [21].

### 2.4. Data Analysis

Responses from household caregivers who remained in the post-intervention survey were analyzed to understand caregivers’ attitudes toward recommended feeding and the practice of dietary diversity. Effects on child nutrition were evaluated for children who attended both pre- and post-surveys. The chi-square test was performed for categorical variables to test background differences between treatment groups: household population, SES, place of delivery, and child sex, statistically. Mann–Whitney U test was used for the continuous variable of child age.

To assess the variable changes per treatment group at the pre- and post-intervention periods, the mean differences of household caregiver situation (DDS and attitude score) and child nutritional status (HAZ, WAZ, and WHZ) were tested using the Wilcoxon signed-rank test for matched pairs. In contrast, multiple linear regression (MLR) was performed to understand the linear relationship between the treatment groups and dependent variables. The differences (post- and pre-intervention) for household caregiver situation and child nutritional status were set as outcomes or dependent variables, and the independent variable was the treatment group. The effect of the intervention was evaluated with and without adjustments for background covariates showing *p*-values less than 0.2 in baseline characteristics, along with pre-intervention scores of the outcome. All statistical analyses were performed using STATA 14 (StataCorp LLC, College Station, TX, USA). Adjusted coefficients with the *p*-value were reported. *p*-values less than or equal to 0.05 were considered statistically significant. QGIS (3.14, 64 bit) (Open Source Geospatial Foundation, available online: qgis.osgeo.org) was used to create the study site map, and world countries were drawn using Natural Earth (1:50 m Cultural Vectors) (available online: www.naturalearthdata.com). Community boundaries were based on the GADM database (available online: gadm.org).

### 2.5. Ethical Consideration

This study was approved by the KEMRI Scientific Ethics Review Unit (SERU) (KEMRI SERU No. 3570) and the Institutional Review Board of the Institute of Tropical Medicine, Nagasaki University (IRB # 171207184-2).

## 3. Results

### 3.1. Participants Characteristics

The study was conducted in two villages in Kwale county, Kenya (Figure 1). According to the HDSS registration, the villages had 662 eligible households for nutritional education (Figure 2). After the trial process, a total of 368 household caregivers (control vs. intervention: 187 vs. 181) and 180 children (113 vs. 67) met the eligibility criteria for the analysis (Table 1). Between the groups, differences in household population (*p* = 0.09), SES (*p* = 0.43), religion (*p* = 0.40), and place of delivery (*p* = 0.66) were not significant. Additionally, there was no significant difference in child characteristics in age (*p* = 0.18) and sex (*p* = 0.72). 

The total drop-out cases included participants who were absent at the post-intervention survey and had missing and contradictive data. Significant differences in household characteristics listed in Table 1 and child sex were not observed between participants who dropped out and completed both pre- and post-intervention surveys. 

### 3.2. Educational Effects

Table 2 shows within-group improvements in household caregiver situations for both control and intervention groups. From pre- to post-intervention, the means of DDS increased similarly in both groups, 1.97 ± 0.45 in the control and 1.94 ± 0.53 in the intervention group (*p* < 0.01). Likewise, the means of the attitude score by group were significantly improved (control improved: 0.46 ± 0.15; *p* < 0.01; intervention improved: 0.49 ± 0.41; *p* < 0.01). Concerning child nutritional statuses, the means of HAZ, WAZ, and WHZ for each group had no evident changes between pre- and post-intervention (Table 2). The differences between pre- and post-intervention in attitude towards recommended feeding within the group are shown in Table S2 (Supplementary Materials).

In Table 3, the difference in household DDS (β = 0.54; *p* < 0.01) and attitude score (β = 0.29; *p* < 0.01) between the groups demonstrated significant educational effects on the intervention group in the adjusted analysis, although no differences were shown in unadjusted modeling. No educational effects between the treatment groups were identified in child nutritional status. Regarding the factors associated with the difference, pre-intervention scores of each outcome negatively related to each corresponding difference (*p* < 0.01). Besides, significant decreases in DDS were observed in the household with the population equal to or more than five members (β = −0.46; *p* < 0.01) and child age (β = −0.02; *p* < 0.01).

## 4. Discussion

The results indicate a significant positive impact of nutritional education with the help of CHWs and SNUs (i.e., Nyumba Kumi) on household caregivers’ attitudes toward recommended feeding and practices of dietary diversity (Table 3). These improvements can be explained by Shi and Zhang’s review on educational intervention for feeding practice, which identified four critical elements for successful interventions [22]. Indeed, our tailored education with SNUs met the first essential element of cultural sensitivity, accessibility, and integration with local resources. The second key element, effective interpersonal communication, was satisfied with our educational strategy of home visit follow-ups. Thirdly, SNUs matched the required key element of community member involvement. Lastly, enrollments of CHWs in education met the fourth essential element, which recommended the use of existing healthcare services.

Furthermore, the success of this study could be better explained by the use of SNUs. It is well known that getting advice from someone known and who is knowledgeable about appropriate feeding practices is more likely to lead to the desired behavior changes of household caregivers [13,23]. In this study, household caregivers had the opportunity to listen to knowledgeable advice from close known community members in the SNU. Recently, the Kenya government adapted the SNU framework or “Nyumba Kumi” for COVID-19 control, which requires flexible and quick responses in local settings. This government action took advantage of the usefulness of using SNUs across the country [24].

While within-group improvements in DDS and attitude scores were observed for both the control and intervention groups (Table 2), the reason could not be explained in our study. These improvements might be due to the effects of the difference in the agricultural seasonality according to the schedule for pre- and post-intervention data collection considering the reports from other studies [25,26,27]. However, it is difficult to conclude that the improvements were due to the seasonality because households in the areas grow several crops for cashing regardless of the season in the study areas. Although neither the control nor intervention groups immediately improved child nutritional status, studies have demonstrated that educational interventions require a longer duration to observe a significant impact on child nutrition [28,29]. Therefore, successfully improved attitudes and practices of household caregivers should lead to enhancements in child nutrition status in the long term. Regrettably, this study was unable to observe such long-term effects on child nutrition because of the limit of the study period, although we observed the improvement of DDS and attitude scores. We need to extend the study period to have positive associations between dietary diversity and HAZ shown in the previous study [30]. Moreover, households with more than five members had a smaller difference in dietary diversity though it was not detected in the attitude score. It may be challenging to increase dietary diversity with the limited household budget for larger families regardless of how much nutritional information they receive; therefore, educational intervention combined with other supports is required to promote behavior changes.

## 5. Conclusions

The results revealed that the interventions using CHWs and Nyumba Kumi (SNUs) were useful to improve dietary diversity and caregivers’ attitudes toward recommended feeding; however, we could not identify the effect on child nutritional status in the short observation period of the study. Similar small neighborhood frameworks with CHWs can be expected to bring positive effects in other regions where similar problems of child nutrition remain.

## Figures and Tables

**Table 1:** Baseline characteristics in the control and intervention groups.

Characteristic	Control	(%)	Intervention	(%)	*p*-Value
Household	*n* = 187		*n* = 181		
Household population					0.09
<5 members	34	(18.2)	46	(25.4)	
≥5 members	153	(81.8)	135	(74.6)	
Socioeconomic status					0.43
Lower	105	(56.2)	109	(60.2)	
Higher	82	(43.8)	72	(39.8)	
Religion					0.40
Islam	162	(86.6)	162	(89.5)	
Other	25	(13.4)	19	(10.5)	
Place of delivery					0.66
Home	60	(32.1)	62	(34.3)	
Facility	127	(67.9)	119	(65.8)	
Child	*n* = 113		*n* = 67		
Sex					0.72
Male	55	(48.7)	35	(51.5)	
Female	58	(51.3)	33	(48.5)	
Age					0.18
Mean age ^a^ (month)	26.41 ± 9.98		24.46 ± 11.30		

^a^: mean ± standard deviation and *p*-values from the Mann–Whitney U test.

**Table 2:** Differences between pre- and post-intervention in household diet practices and child growth.

Variable	Control				Intervention			
	Pre-	Post-	Δ ^a^	*p*-Value ^b^	Pre-	Post-	Δ ^a^	*p*-Value ^b^
Household	*n* = 187				*n* = 181			
DDS ^c^	4.18 ± 1.81	6.15 ± 1.36	1.97 ± 0.45	<0.01	4.97 ± 1.95	6.91 ± 1.42	1.94 ± 0.53	<0.01
Attitude score	7.86 ± 1.25	8.32 ± 1.10	0.46 ± 0.15	<0.01	8.15 ± 1.09	8.64 ± 0.68	0.49 ± 0.41	<0.01
Child	*n* = 113				*n* = 67			
HAZ	−1.56 ± 1.25	−1.55 ± 0.89	0.01 ± 0.36	0.06	−1.30 ± 1.62	−1.51 ± 1.16	0.21 ± 0.46	0.79
WAZ	−1.14 ± 1.10	−1.04 ± 0.81	0.10 ± 0.29	0.36	−0.89 ± 1.13	−0.97 ± 0.88	0.08 ± 0.25	0.36
WHZ	−0.44 ± 1.18	−0.22 ± 0.94	0.22 ± 0.24	0.22	−0.24 ± 1.26	−0.16 ± 1.20	0.08 ± 0.06	0.66

^a^: mean differences between pre- and post-intervention within the group; ^b^: mean changes within the group using the Wilcoxon signed-rank test; ^C^: dietary diversity score.

**Table 3:** The effects ^a^ of educational intervention and participants demographic variables for household diet practice and child growth between pre- and post-intervention.

Variable	DDS ^b^ (*n* = 368)				Attitude Score (*n* = 368)				HAZ ^c^ (*n* = 180)				WAZ ^d^ (*n* = 180)				WHZ ^e^ (*n* = 180)			
	Unadjusted		Adjusted		Unadjusted		Adjusted		Unadjusted		Adjusted		Unadjusted		Adjusted		Unadjusted		Adjusted	
	β	*p*-Value	β	*p*-Value	β	*p*-Value	β	*p*-Value	β	*p*-Value	β	*p*-Value	β	*p*-Value	β	*p*-Value	β	*p*-Value	β	*p*-Value
Group																				
Control (reference)																				
Intervention	−0.04	0.86	0.54	<0.01	0.02	0.89	0.29	<0.01	−0.22	0.24	−0.04	0.79	−0.19	0.11	−0.08	0.38	−0.14	0.46	−0.05	0.72
Adjusted variable Pre-intervention score of outcome																				
	−0.8	<0.01	−0.79	<0.01	−0.89	<0.01	−0.90	<0.01	−0.65	<0.01	−0.62	<0.01	−0.46	<0.01	−0.46	<0.01	−0.62	<0.01	−0.61	<0.01
Household population																				
<5 members (reference)																				
≥5 members	−0.81	<0.01	−0.46	<0.01	0.03	0.85	−0.02	0.86	0.2	0.37	0.04	0.79	0.09	0.53	−0.03	0.8	−0.14	0.47	−0.05	0.76
Child mean age (month)	−0.05	<0.01	−0.02	<0.01	−0.00	0.69	−0.00	0.25	0.04	<0.01	0.01	0.04	0.01	0.21	−0.00	0.30	−0.02	0.04	−0.02	0.01

^a^: multiple linear regression (MLR) estimated the effects of the educational intervention and participants demographic variables by the difference of the mean change from pre- to post-intervention. The effect of the intervention was measured without and with adjustments for background covariates showing *p*-values <0.2 in baseline characteristics and pre-intervention scores of outcome; ^b^: dietary diversity score; ^c^: height-for-age z-score; ^d^: weight-for-age z-score; ^e^: weight-for-height z-score.

## Supplementary Materials

The following are available online at https://www.mdpi.com/1660-4601/17/19/7269/s1, Table S1: Nine question items used to assess caregivers’ attitudes, Table S2: Differences between pre- and post-intervention in attitude towards recommended feeding within the group, respectively.
