# Supplementary material for: Improvement of Dietary Diversity and Attitude toward Recommended Feeding through Novel Community Based Nutritional Education Program in Coastal Kenya—An Intervention Study
Source: Int J Environ Res Public Health. 2020 Oct 5;17(19):7269. doi: 10.3390/ijerph17197269 (PMC7579186; doi:10.3390/ijerph17197269)
Supplement: Supplementary file 1 [file ijerph-17-07269-s001.pdf]

Table S1. Nine question items used to assess caregiver attitude.

Item	Question items
1	Colostrum should be given to a newborn
2	Early initiation of exclusive breastfeeding is beneficial to a newborn within one hour of birth
3	Breastmilk contains sufficient water
4	Babies should be breastfed without being given any other food including water, up to six months
5	Babies should be breastfed on demand, at all times including night time
6	Complementary foods should be introduced at six months
7	After introduction of complementary foods, there should be continued breastfeeding
8	Breastfed Children 6-23 months should be fed more than four times a day
9	Children over six months should be fed a variety of foods each day

International Journal of Environmental Research and Public Health

Table S2. Differences between pre- and post-intervention in attitude towards recommended feeding within the group

Question Items	Control (n = 187)					Intervention (n = 181)				
	Pre-		Post-		P-value[a]	Pre-		Post-		P-value[a]
	n	(%)	n	(%)		n	(%)	n	(%)	
Colostrum					0.17					<0.01
Agree	167	89.3	175	93.6		154	85.1	179	98.9	
Disagree	20	10.7	12	6.4		27	14.9	2	1.1	
Early initiation of BF[b]					0.85					0.29
Agree	174	93.1	172	92.0		161	89.0	167	92.3	
Disagree	13	6.9	15	8.0		20	11.0	14	7.7	
Water in Breastmilk					0.22					0.68
Agree	157	84.0	166	88.8		168	92.8	165	91.2	
Disagree	30	16.0	21	11.2		13	7.2	16	8.8	
EBF[c]					0.29					0.22
Agree	173	92.5	179	95.7		172	95.0	177	97.8	
Disagree	14	7.5	8	4.3		9	5.0	4	2.2	
Timing of BF					1.0					0.63
Agree	185	98.9	186	99.5		178	98.3	180	99.5	
Disagree	2	1.1	1	0.5		3	1.7	1	0.5	
Beginning of CF[d]					0.58					0.58
Agree	181	96.8	178	95.2		175	96.7	172	95.0	
Disagree	6	3.2	9	4.8		6	3.3	9	5.0	
Continued BF					1.0					0.45
Agree	183	97.9	184	98.4		176	97.2	179	98.9	
Disagree	4	2.1	3	1.6		5	2.8	2	1.1	
Meal frequency					<0.01					<0.01
Agree	122	65.2	153	81.8		127	70.2	171	94.5	
Disagree	65	34.8	34	18.2		54	29.8	10	5.5	
Dietary diversity					<0.01					0.08
Agree	127	67.9	163	87.2		165	91.2	174	96.1	
Disagree	60	32.1	24	12.8		16	8.8	7	3.9	

a: Proportion differences between pre- and post-intervention within the group using McNemar test; b: Breastfeeding; c: Exclusive breastfeeding; d: Complementary feeding

Question Items: Colostrum = Colostrum should be given to a newborn; Early initiation of BF = Early initiation of exclusive breastfeeding is beneficial to a newborn within one hour of birth; Water in Breastmilk = Breastmilk contains sufficient water; EBF = Babies should be breastfed without being given any other food including water, up to six months; Timing of BF = Babies should be breastfed on demand, at all times, including night time; Beginning of CF = Complementary foods should be introduced at six months; Continued BF = After the introduction of complementary foods, there should be continued breastfeeding; Meal frequency = Breastfed Children 6–23 months should be fed more than four times a day; Dietary diversity = Children over six months should be fed a variety of foods each day
